# Intelligent Protein Design and Molecular Characterization Techniques: A Comprehensive Review

**DOI:** 10.3390/molecules28237865

**Published:** 2023-11-30

**Authors:** Jingjing Wang, Chang Chen, Ge Yao, Junjie Ding, Liangliang Wang, Hui Jiang

**Affiliations:** State Key Laboratory of NBC Protection for Civilian, Beijing 102205, China; wjj18811039053@163.com (J.W.); chenchang15@mails.ucas.ac.cn (C.C.); bzyaoge@163.com (G.Y.)

**Keywords:** intelligent protein design, protein characterization techniques, sequence characterization, structural characterization

## Abstract

In recent years, the widespread application of artificial intelligence algorithms in protein structure, function prediction, and de novo protein design has significantly accelerated the process of intelligent protein design and led to many noteworthy achievements. This advancement in protein intelligent design holds great potential to accelerate the development of new drugs, enhance the efficiency of biocatalysts, and even create entirely new biomaterials. Protein characterization is the key to the performance of intelligent protein design. However, there is no consensus on the most suitable characterization method for intelligent protein design tasks. This review describes the methods, characteristics, and representative applications of traditional descriptors, sequence-based and structure-based protein characterization. It discusses their advantages, disadvantages, and scope of application. It is hoped that this could help researchers to better understand the limitations and application scenarios of these methods, and provide valuable references for choosing appropriate protein characterization techniques for related research in the field, so as to better carry out protein research.

## 1. Intelligent Design for Protein Molecules

**Figure 1:**
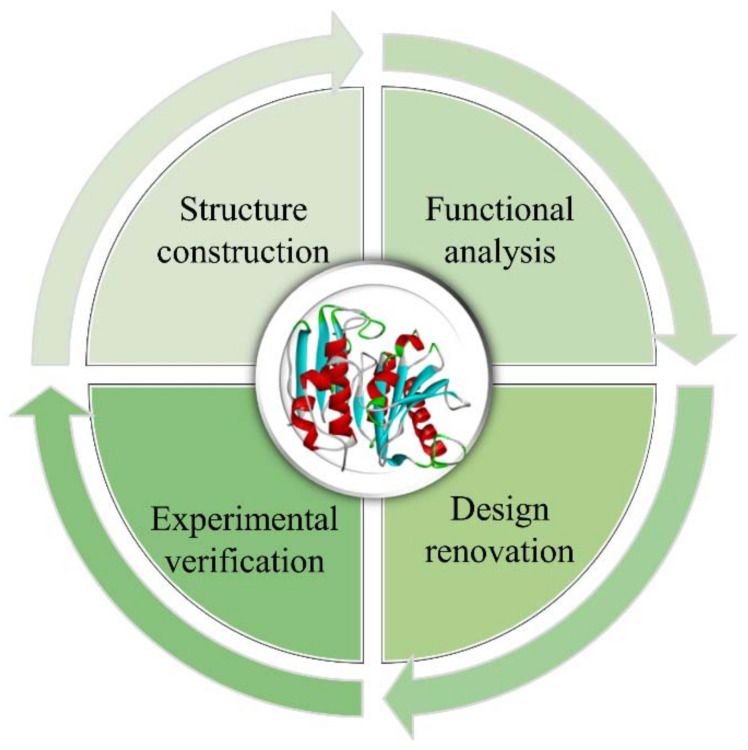
The flowchart for protein design.

Protein molecular design refers to the comprehensive use of multidisciplinary techniques to obtain novel proteins with better target performance than natural proteins based on the structure–function relationship of proteins. This process mainly involves establishing a structural model of the target protein, studying the structure–function relationship, proposing a reasonable design and renovation plan, and further modifying the design through experimental verification, which often requires multiple iterations to achieve the desired purpose (Figure 1) [1]. The main types of protein structural designs include: (1) Minor, (2) Moderate, (3) Major modifications, which can be described, in order, as follows: (1) Artificially modifying amino acid (AA) residues of natural proteins with known structures to investigate and improve their function and properties, (2) Splicing and assembling protein structural domains from different sources to obtain protein molecules with new functions through the transfer of the corresponding functions, (3) Designing entirely new proteins with specific spatial structures and functional properties from scratch [1,2].

Early work in protein design focused on redesigning helical bundles [3], using strategies designed to generate specific hydrophobic/hydrophilic patterns to control the protein folding process without predicting specific side chain orientations [4,5,6]. In 1997, protein structure design methods were gradually extended to irregular geometries to increase the diversity and variability of backbone structures in protein design [7]. For example, RosettaDesign, a universal computing protocol, was used to predict the low-freedom energy sequences of nine natural protein backbones. Comparing the NMR structure of the predicted sequence with that of the natural protein, showed that RosettaDesign could reliably identify the amino acid sequence of the protein backbone [8]. In 2003, the Baker Lab continuously iterated between sequence design and structure prediction to break the existing topologies of protein redesign, obtain novel protein folding structures, and produce the Top7 α/β topology [9]. However, the early exploratory efforts targeting computational protein design suffered from a small range of structural modifications, low success rates, and ineffective results. They relied on cyclic iterative experimental screening, which resulted in significant consumption of human, material, and time resources.

**Figure 2:**
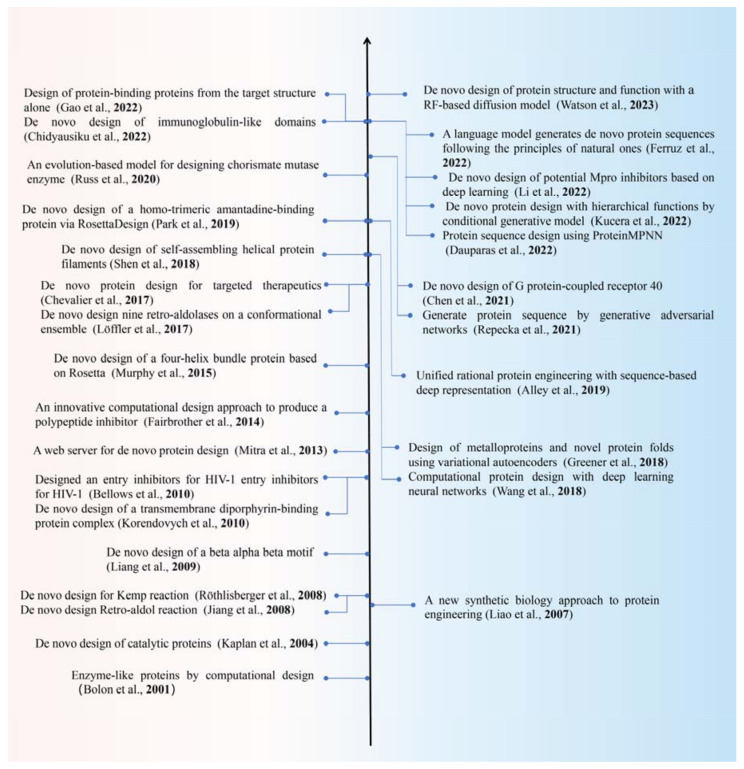
The summary of the classic examples of de novo protein design [14,15,16,17,18,19,20,21,22,23,24,25,26,27,28,29,30,31,32,33,34,35,36,37,38,39,40,41]. The left and right of the figure show model-based and data-driven examples, respectively.

In recent years, the updated optimization of artificial intelligence algorithms, increasing arithmetic power of computer hardware, and massive expansion of experimental protein structure data have created favorable infrastructure for intelligent protein design, resulting in many remarkable results (Figure 2). In 2019, Ingraham et al. [10] introduced a protein generation model based on a graphical representation of the three-dimensional (3D) structure of proteins, which improves both computational speed and reliability compared to traditional neural-network-based approaches due to its ability to exploit the spatial localization of dependencies in the molecular structure. In 2020, Strokach et al. [11] developed a deep graph neural network called ProteinSolver that was trained to precisely design sequences that were folded into a predetermined shape. Deep graph neural networks can rapidly design specific novel protein sequences, which are difficult to achieve using traditional computational design methods. In 2021, Anishchenko et al. [12] from the Baker Lab developed a deep neural network hallucination method, using trRosetta, which is trained in protein structure prediction and has the capability to capture protein sequences and structural information as a background network. This method generates new protein sequences with specific functions by “inducing” random sequences from the input. This facilitates an exploration of all possible protein structure spaces that is credited to the ability of deep learning to process large datasets. The following year, Wang et al. [13] from the same group developed a deep learning method based on hallucination and inpainting to enable the construction of protein binding and catalytic functional sites without pre-specifying backbone folding or secondary structure.

The core of intelligent protein design involves establishing a relationship between structure and function. Therefore, the prediction of protein structure and function by artificial intelligence algorithms is also a key aspect of protein design, apart from in the above-mentioned intelligent protein design methods that directly modify protein structure to target the desired performance. AlphaFold2 [42], which has made great progress on the “protein structure prediction” problem that has plagued the academic community for five decades, has predicted structures covering 98.5% of the human proteome [43]; similarly, these data will provide a transformative impact on the intelligent design of proteins with specified functions. In February 2022, Bileschi et al. [44] used a dataset constructed from the Protein Families Database (Pfam) to train a neural network (called ProtCNN) to functionally classify protein sequences to achieve a 200-fold increase in speed, and a 9-fold reduction in error, compared to the traditional BLASTp method. This advancement in functional prediction provides a powerful tool for accelerating the intelligent design of proteins.

It is evident that the latest advances in artificial intelligence algorithms (especially deep learning technology) can boost the overall intelligent protein design process by assisting protein structure modification and structure and function prediction [10,11,12,13,42,43]. Structural characterization of protein molecules is a crucial part of the intelligent protein design process. The ability to represent protein structures in a comprehensive, accurate, and efficient manner in a machine-recognizable language or vector is essential for the success of downstream intelligent protein design tasks using intelligent algorithms. This review systematically described various protein characterization techniques and representative applications used in intelligent protein design and discussed their advantages, disadvantages, and application areas. We hope to provide a valuable reference for scholars to conduct relevant research in this field.

## 2. Examples of Applications for Intelligent Protein Design

Artificial intelligence has been used in many applications in the field of protein engineering; including protein structure, function, thermal stability [45,46,47], and stereoselectivity prediction [48,49]; owing to its high accuracy, fast computational speed, and independence from protein structure and function information compared with earlier protein design methods. Various deep learning algorithms and natural language processing (NLP) techniques based on deep learning were successfully used in numerous applications, apart from classical machine learning algorithms (support vector machines, decision trees, Gaussian regression, and so on) [50,51,52,53,54]. The following section focuses on three recent successful cases in protein structure prediction, function prediction, and de novo protein design to systematically analyze the advantages of artificial intelligence algorithms applied in protein engineering.

### 2.1. Protein Structure Prediction

Protein structure prediction is a critical step in the intelligent design of proteins and is a fundamental scientific problem in the field of protein computation. This problem can be traced back to the famous statement made by Christian B. Anfinsen (the Nobel laureate in chemistry in 1972), that the AA sequence of a polypeptide chain contains all the information about its 3D structure [55]. Currently, experimental techniques for obtaining 3D protein structures include X-ray crystallography [56], nuclear magnetic resonance (NMR) [57], and cryo-electron microscopy (cryo-EM) [58]. There are only about 205,000 experimentally resolved protein structures in the Protein Data Bank (PDB) as of June 2023 [59], while the UniProt database contains over 250 million sequences [60]. This means that the number of proteins with known sequences is more than 1200 times greater than the number of experimentally resolved protein structures. In contrast, the number of known protein sequences was only 160 times that of the experimentally resolved protein structures in 2011 [61]. It is evident that the number of protein structures solved is far lower than the total number of protein sequences.

To address this problem, the academic community has been organizing the critical assessment of protein structure prediction (CASP) competitions since 1994, which has greatly promoted the development of computational methods for protein structure prediction. For example, I-TASSER [62] represents a homology modeling approach that uses threading to predict structures and has won multiple championships in the CASP. In 2020, AlphaFold2 [42], developed by DeepMind, won CASP14 by a landslide using the transformer algorithm. In 2022, DeepMind released the AlphaFold protein structure library, AlphaFold DB [63], demonstrating the dominance of the AlphaFold tool for protein structure prediction. In addition, RoseTTAFold [64], developed by Baker Lab, achieved considerable prediction accuracy at CASP14, ranking only behind AlphaFold2. Novel artificial intelligence-driven protein folding prediction tools such as AlphaFold2 and RoseTTAFold provide powerful drivers for rapid and accurate protein structure prediction and subsequent protein design modifications [65,66,67,68,69,70,71,72,73]. Many studies were conducted using them to further improve the accuracy and speed of protein structure predictions. Table 1 summarizes the methods, models, and functions of relevant studies.

The advent of AlphaFold2 and RoseTTAFold has increased the accuracy of protein structure prediction to a new level. However, these methods are not effective at predicting the structure of orphan proteins because of the lack of homologous proteins. In October 2022, Chowdhury et al. [54] proposed an end-to-end recurrent geometric network computational model named RGN2 that predicts the structure of orphan proteins with better accuracy than AlphaFold2 and RoseTTAFold. It uses the protein language AminoBERT to parse the potential structural information of orphan proteins, and its computational efficiency is 106 times faster than that of AlphaFold2. In November of the same year, Wang et al. [52] proposed a single-sequence protein structure prediction algorithm called trRosettaX-Single. The algorithm integrates sequence embeddings from the Transformer protein language model into a knowledge-distillation-enhanced multiscale network to predict two-dimensional geometric structures between residues. Then, the three-dimensional structure is reconstructed using an energy minimization approach, which improves the accuracy and efficiency of orphan protein structure prediction.

In addition to protein monomer structure prediction, multimer structure prediction has also been studied recently. In October 2021, the DeepMind team developed AlphaFold-Multimer [74], with innovative multi-chain feature extraction and symmetric replacement modules based on AlphaFold2. It achieved prediction accuracies of 67% and 69% at the contact interface of heterologous and homologous multimers, respectively. In September 2022, Tang et al. [75] proposed the first MSA pairing algorithm, ColAttn, which combines the outputs of protein language models into a joint MSA form to identify paired homologs from single chains using the attention score in the MSA Transformer, making it demonstrate the best structure prediction accuracy on heterodimers. Meanwhile, Uni-Fold v.2.0.0 [66], released by DP Technology, also added a protein multimer structure prediction function. The tool is modeled on the model AlphaFold-Multimer architecture and modifies and optimizes the model details, achieving a two-fold increase in speed and accuracy. In addition, Zhang Yang’s lab proposed the DMFold-Multimer [73], which combines DeepMSA2 for searching homologous sequences from large-scale genomic and metagenomics databases with AlphaFold2-Multimer’s structure model generator, leading to the champion of the protein complex structure prediction project in the CASP15. However, these studies were mainly constrained by the limited number of multimer structures used for training and the lack of accurate characterization of multimer clustering relationships. This provides limited prediction accuracy and few structure predictions of protein–ligand complexes. In conclusion, the structure prediction of protein monomers with homologs was basically solved with the advent of AlphaFold2 and Uni-Fold v.2.0.0. The accuracy of the structural predictions for orphan proteins and multimers requires improvement. Moreover, structural prediction research on protein–ligand complexes is sparse, and mainly relies on docking and dynamic simulations to predict protein–ligand binding patterns. The direct prediction of protein–ligand complex structures by artificial intelligence-based methods would receive significant attention from scholars with the increase in experimentally resolved protein–ligand complex structures, the development of protein complex characterization methods, and the further improvement of computer performance.

### 2.2. Protein Function Prediction

The primary sequence of a protein determines its high-level structure that determines its function according to the golden rule of sequence–structure–function correspondence. Thus, the protein sequence ultimately determines protein function. A deep understanding of the relationship between protein sequence and function enables the rapid localization of novel protein functions that facilitates de novo protein design by direct sequence modification. The advent of low-cost and efficient sequencing technologies has driven rapid growth in the number of protein sequences [76,77]. The UniProtKB database contains over 200 million sequences, with only approximately 0.25% manually annotated by March 2022 [78]. Determining the relationships between sequences and functions has become a critical issue in protein design with the growing number of protein sequences.

In 2020, Hippe et al. [79] proposed the ProLanGO2 method that follows the design principles of natural language translation and uses sequence-based recurrent neural networks for protein function prediction. Its prediction performance is comparable to that of other sequence-based methods, and even the network-based method NetGO2.0. ProLanGO2 has proven its potential for protein function prediction by converting protein function prediction into natural language translation. In 2021, Gligorijević et al. [80] proposed a graphical convolutional network model, DeepFRI, which combines deep learning with more available sequence information to substantially improve protein function prediction. In the same year, Yong et al. [81] proposed an automated protein function prediction method based on graph neural networks, DeepGraphGO, which significantly outperformed many state-of-the-art methods by fully combining protein sequences and higher-order protein network information. In 2022, Zhang Yang’s lab established a unified and efficient multi-domain protein structure and function prediction platform, I-TASSER-MTD, by integrating methods developed by his lab in recent years. This includes protein sequence structural domain delineation, deep learning spatial geometric constraint prediction, single-domain structure modeling, multi-domain structure assembly, and structure-based function annotation to achieve fully automated multi-domain protein structure and function prediction from protein sequences [51].

Various types of information were used for automated prediction of protein function in addition to sequence-based methods for intelligent prediction, such as domain-based prediction [82,83,84], homologous-protein-based functional transfer [85,86,87], and protein-network-dependent methods [88,89,90]. However, there is a lack of functional sites, homologous proteins, or biological network information for newly sequenced or less studied proteins. Therefore, in future protein function prediction, we can focus on three key aspects. Firstly, we need to accurately characterize the relationship between protein structure and function to enhance the overall performance of protein function prediction. Secondly, we can use correlations between different functions to aid in the precise localization of protein functions for multifunctional proteins. Lastly, we can integrate protein structural features, global and local sequence features, and genomic contextual environmental features to achieve accurate protein function prediction.

### 2.3. De Novo Protein Design

The emergence of de novo protein design can be traced back to the 1980s, when DeGrad et al. [91] made a preliminary attempt at protein design and successfully constructed stable four-stranded helix bundles using rule-based heuristics. In the late 1990s, Dahiyat et al. [7] pioneered the design of AA sequences using an automated optimization approach with the development of molecular mechanics energy functions, AA side-chain conformational libraries, and optimization algorithms.

The automatic design method based on energy functions is not limited by the type of main chain structure compared with the purely heuristic design method. Furthermore, the specific spatial accumulation between residues and the quantitative calculation of hydrogen bond interactions improves the success rate of the design. In the 21st century, Baker first designed protein folding that does not exist in nature, leading to the de novo design of protein backbones. In 2008, Baker proposed an inside-out protein design strategy to artificially create several non-natural enzymes (such as Diels–Alder synthase [92], Kemp eliminase [14], and Aldolase [15]) through theoretical computational design.

In recent years, algorithms emerging from the de novo design of proteins were gradually applied to the structural-functional remodeling of natural proteins. In 2015, the David Lab group re-engineered formaldehyde polymerase (FLS) to catalyze the polymerization of formaldehyde using a specific natural benzaldehyde lyase (BAL) unearthed from a database and employing Foldit and RosettaDesign tools [93]. Further modification of the FLS design increased its activity 4.7-fold in 2021. This makes it a key enzyme in the in vitro pathway converting inorganic carbon to organic carbon in the synthesis of starch from CO_2_ [94]. Most of the above studies used energy functions as indicators for protein design evaluation or tools, such as Foldit and RosettaDesign, for de novo protein design, collectively referred to as model-based de novo protein design. Classic de novo protein design examples are presented in Figure 2.

Data-driven approaches to de novo protein design (including structural data and massive protein sequences) have also emerged in recent years [32,33,34,35,36,37,38,39,40,41,95] along with the wave of big data and artificial intelligence development, the development of high-throughput data collection methods, and the accumulation of available data. Liu and co-authors made a significant contribution to the development of data-driven protein design methods [96]. The authors constructed the SCUBA model for the de novo design of protein backbone structures, using neural network energy functions and the statistical energy model ABACUS. This method is critical for designing AA sequences for a given backbone structure, and it is the only fully experimentally validated method for the de novo design of proteins besides RosettaDesign. However, this approach to sequence design by optimizing the energy function has limited success rates and computational efficiency. The Baker Lab proposed a multi-stranded and symmetry-aware model architecture, ProteinMPNN, which generates sequences that fold more reliably and accurately into the natural protein backbone than the original natural sequences [40]. This tool significantly improves computational efficiency compared to physically based methods, such as Rosetta. It is widely used in protein design, owing to its high design success rate, low time consumption, and applicability to almost all protein sequence designs [97,98]. In addition, Baker Lab also constructed a versatile protein design framework based on an RF-based diffusion model, RFdiffusion, which enables de novo binder design and the design of higher-order symmetric architectures [41].

De novo protein design using computational design has entered an unprecedented era, wherein the structural and functional design of increasingly complex proteins would be possible with the continuous iterative optimization of energy functions, main chain design, and side chain optimization. A recent review by Ovchinnikov and Huang described how structural information can replace traditional backbone design, side-chain optimization, and energy functions [99]. Huang used structural features of AA neighbors to construct “higher-order soft potential energy functions” [100]. Comparing traditional methods against deep learning methods is an important issue in the anticipation of new methods.

## 3. Macromolecular Characterization Techniques and Their Application in Intelligent Protein Design

Molecular characterization refers to measuring molecular properties in a certain aspect that is either the basic physical and chemical properties of molecules, or numerical indicators or vectors derived from the molecular structure using various algorithms to describe the structural information of different layers of molecules [101]. They can be divided into small and macromolecular characterizations depending on the size of the molecular system. The threshold and difficulty of characterizing biomacromolecules is significantly higher compared with the characterization techniques of small molecules, owing to their higher molecular mass and higher structural complexity. Protein intelligence design involves extracting and encoding the structural features of biological macromolecules, such as DNA, proteins, and RNA, as quantitative vectors. These vectors are then used for machine learning-based modeling tasks, including predicting protein binding regions [102,103,104], functions [105,106,107], physical and chemical properties [108,109,110,111], and more. This review characterized techniques for protein macromolecules as divided into four categories, according to the degree of description for the structure information: (1) Characterization based on traditional molecular descriptors, (2) Sequence-based characterization, (3) Structure-based characterization, (4) Hybrid sequence–structure-based characterization. The subsequent sections focus on these four aspects of macromolecular characterization techniques and the corresponding application cases, as well as systematically analyzing the characteristics, advantages, and limitations of each characterization method.

### 3.1. Characterization Based on Traditional Molecular Descriptors

In the early years, computer development was relatively delayed and hardware standards were low. Traditional classical descriptors were widely used for crude characterization of protein macromolecules, owing to their simplicity, ease of understanding, and low arithmetic requirements. These traditional descriptors typically quantitatively describe the intrinsic properties of a macromolecule based on its molecular composition and physicochemical properties, including the frequency of AA occurrences in the protein composition [112], the isoelectric point used to determine the charge of the protein in different pH solutions [113,114], the hydrophilicity and hydrophobicity (which plays a major role in maintaining protein conformation) [115,116,117], the absolute charge of the protein [118], the sequence entropy to reflect the conservation and variability of the protein AA sequence [119], the sequence length and molecular weight to reflect the protein length and size [120], the solvent accessible surface area (SASA) [121,122] to indicate the degree of AA exposure of a protein, and the dipole moment [123,124] (used to determine the spatial conformation of a molecule), and so on. Characterization methods can be divided into two categories: sequence-based and structure-based. The characteristics, categories, and applications of each traditional descriptor representation are discussed in detail in Table 2.

Early traditional descriptors in intelligent protein design were mostly used in studies of protein–macromolecule interactions, protein–small molecule interactions, and protein functional site predictions [125,126,146,147,148,149]. Liu et al. [128] proposed a model called aPRBind to predict the binding residues of RNA in proteins by convolutional neural networks, that integrates the sequence features based on the spatial neighbor-based position-specific score matrix (SNB-PSSM) and structural features (including residue-kinetic properties and residue-nucleotide propensities), based on the I-TASSER model, to achieve superior predictive performance compared to other advanced methods. However, the best sensitivity, specificity, accuracy, and Mathew’s correlation coefficient were 0.65, 0.82, 0.74, and 0.48, respectively, indicating that there is still room for improvement in protein–RNA binding site prediction. Traditional descriptors do not provide a comprehensive characterization of the global information of an RNA/protein. Therefore, the accuracy of more complex prediction tasks (such as functional sites) requires improvement. Consequently, these methods are inappropriate for more complex protein design.

### 3.2. Sequence-Based Characterization

Protein sequence determines the three-dimensional structure. Therefore, the protein sequence contains advanced structural information. Most protein-related studies have employed sequence information to characterize the proteins when protein structures were difficult to resolve and computing power was insufficient. One-hot and K-mer characterization methods were used extensively, owing to their simplicity and ease of understanding, low computational effort, and high efficiency [150,151,152,153,154]. In addition, protein sequence characterization methods such as word2vec [155,156], seq2vec [157], BioVec [158], doc2vec [159], and N-gram [160,161] were proposed and applied based on the intrinsic similarities between protein sequences and natural languages.

A large variety of NLP models have emerged with a profound impact on the study of intelligent protein design following advances in sequencing technology, the development of deep learning algorithms, and significant improvements in computing power. In 2017, Google released transformers based on the attention model that started a new era of NLP [162]. This greatly improved the performance of various tasks, including clinical diagnosis, image recognition, and protein–ligand affinity prediction [163,164,165,166,167,168]. Countless adaptations of pre-trained language models have emerged, including the Bidirectional Encoder Representation from Transformers (BERT) based on the transformer encoder structure [169], the Generative Pre-trained Transformer (GPT) and the successors GPT-2 and GPT-3 [170,171,172], the Evolutionary Scale Modeling (ESM) family for predicting protein structure and function (ESM-1b, ESM-MSA-1b, & ESM-1v) [173,174,175], the ProtTrans with the largest training dataset [176], and the ProGen language model [177] that can control protein generation.

Advances in the transformer era inspired several studies to apply the concept of language models to protein design. In February 2019, Yu et al. [161] applied n-gram modeling to generate a probabilistic protein language model. In October 2019, Alley et al. [34] applied a multiplicative long short-term memory network (mLSTM) to learn a language model that predicted protein sequence stability with higher accuracy. In July 2022, Höcker et al. [37] proposed a language model trained on protein space ProtGPT2 to generate new protein sequences according to natural principles. In December 2022, Rives et al. [178,179] found that the ESM2 language model can generate new proteins beyond natural proteins and generate complex and modular protein structures by learning and programming deep grammar.

NLP-based learning models for protein sequence representation have achieved remarkable results in protein design [34,37,161,178,179]. However, there are deep grammatical structural differences between modeling languages and protein representations. It is estimated that a native American English speaker uses approximately 46,200 words on average and multi-word expressions. However, only 20 different AAs are processed in proteins by representation models in a manner similar to a linguistic lemma. Moreover, these language models have relatively high spatial and temporal complexities. For example, the ESM-2 model with 15 billion parameters, requires significant computational time and powerful computing equipment for training.

It is anticipated that NLP models will be further improved by simplification and reducing their dependence on computing devices. New protein characterization methods will be developed to better represent the relationship between protein and natural language. Alternatively, we may see the continued growth of protein sequences and the implementation of quantum computers that will allow protein design models to achieve human-like thinking and precisely achieve the second law proposed by Manfred Reetz: “You get what you designed” [180].

### 3.3. Structure-Based Characterization

Proteins are composed of one or more peptide chains, and the connections and folding patterns of each peptide chain constitute their special three-dimensional spatial structure [181]. The unique spatial structure determines the specific biological functions. In theory, obtaining the structural information of proteins could lead to a better understanding of the relationship between the structure and function of proteins, which could lead to a better intelligent protein design. Therefore, protein intelligent design and functional studies require structural characterization. Structural characterizations can be divided into graph structure-based and geometric structure-based characterizations according to the manner in which they are performed. Graph structure-based characterization methods can be divided into topology and distance-graph-based protein characterization methods.

#### 3.3.1. Graph Structure-Based Characterization

##### Topology Structure-Based Protein Characterization

Topology-based protein characterizations describe AAs based on the atomic linkage indices generated from molecular graphs. These mainly include traditional T-scale and ST-scale topology descriptors, and newer meta-graph and circuit topology descriptors.

In 2007, T-scale was proposed by Tian et al. [107] based on a computer program. generating 67 generic topological descriptors based on 135 AAs. However, these descriptors do not explicitly consider the 3D features of each structure, and they are based only on the strength of the AA linkage table. In 2009, ST-scale proposed by Yang et al. [182] used the 3D information of 167 AAs and PCA based on 827 structural dimensions. The chemical structure of a set of peptides and their analogs can be characterized by describing the position of each AA using eight ST-scale values based on ST-scale.

A meta-graph is a newly proposed graph structure that differs from the traditional network themes or sub-graphs. It captures specific topological arrangements involving interactions and associations between proteins and keywords. Each protein can be described by a series of meta-graphs illustrating its interactions with other proteins and their associations with keywords. Proteins with similar functions often exhibit similar meta-graph representations [183].

Circuit topology is a newly proposed descriptor that theoretically assesses the relationship between contact pairs on the protein backbone and provides information about the protein structure (such as the order of residues and residue contacts). The use of circuit topology to predict the folding rate of proteins has improved pathogenicity prediction of missense mutations [184].

##### Distance Map-Based Protein Characterization

Protein distance graphs can be obtained by calculating the distance between Cα atoms or neighboring residues. A protein of length n can be represented as a *n* × *n* matrix, and descriptor values can then be obtained using matrix decomposition or image processing techniques. The contact graph is a binary graph obtained by setting a distance threshold on the distance graph. Distance and contact graphs have the advantage of rotational or translational invariance of the protein structure and low dimensionality, which makes them computationally efficient. Currently, contact, and distance graphs were extensively used in protein structure prediction methods, such as AlphaFold [185], trRosetta [186], C-I-TASSER [187], C-QUARK [188], DeepFold [189], and so on.

#### 3.3.2. Geometry-Based Characterization

Geometry-based protein characterization is related to indicators representing the structural features of a protein, such as the locations of atoms in space, and the shape and size of the protein. These include point clouds [50], three-dimensional tessellation [190], three-dimensional convolutional neural network (3D-CNN) [191], and GVP-GNN [192].

A point cloud is a set of points representing object-space partitioning and external attributes in the same spatial reference system. It is a group of isolated nodes with a given position in 3D space, called a 3D point cloud [50]. Point clouds are significantly faster than other procedures in terms of data processing. They can be directly processed by rotation and other variable operations, thereby avoiding extension of the data. Currently, point clouds are used in areas involving protein–ligand binding affinity prediction and protein–ligand binding site prediction [50,193].

Three-dimensional tessellations allow graphical representation of proteins by dividing the three-dimensional space into cells with specific properties. Each node represents a cell and any contact between two cells is represented by each edge. A Voronoi diagram is a typical type of tessellation that describes the structure and interactions of proteins and is mostly applied in structural bioinformatics [190]. For example, it is used to estimate the deviation between the predicted and native protein structures [194], and to analyze the structure of protein–protein interactions [195]. An effective programming representation of Voronoi graphs requires quite a complex data structure. The high cost of developing and maintaining these data structures is a notable barrier to fully utilizing this powerful mathematical concept in practice.

The 3DCNN divides 3D space into multiple grids, allowing direct manipulation of atomic positions in space by vowelizing the structure and facilitating the capture of the local microenvironment of the protein structure. Thus, the 3DCNN automatically extracts protein structural features and has powerful structural characterization capabilities that are compatible with the detection of structural patterns, binding pockets, and other important structural features of specific shapes. Li et al. [191] used deep 3D convolutional neural networks to predict the changes in the thermodynamic stability of proteins upon point mutations. Zhao et al. [196] predicted the binding sites of metal ions on RNA by 3DCNN.

The GVP-GNN introduces Geometric Vector Perceptrons (GVPs) and extends the standard dense layer to enable manipulation of a collection of Euclidean vectors [192]. By introducing GVPs, GVP-GNN can incorporate protein 3D structure vectors into GNNs that satisfy rotational translation covariance and conveniently capture spatial neighborhood information to enhance the ability of the GNN to represent proteins. GVP-GNN can also accomplish covariant and invariant representation of biomolecular geometry with lightweight parameters. It is well suited for biomolecules and biomolecular complexes and is expected to be further developed in the field of intelligent protein design.

### 3.4. Hybrid Sequence–Structure-Based Characterization

Protein design often relies on three-dimensional structural data to fully capture the functional information of proteins. It is typically richer than the information provided by sequence data. However, current models predominantly use sequence features, owing to the lack of proper 3D structure characterization methods. Most are computationally expensive and cannot avoid information loss when dimensional reduction is performed. Furthermore, deep learning models may not fully explore the hidden information in high-dimensional data [197]. Consequently, multi-scale representation methods that incorporate sequence and structural information have emerged for protein design. Currently, there is a lack of direct representation methods for multimodal data; therefore, researchers mainly separately use the sequence and structural representation methods described above, and then merge the extracted feature vectors using downstream models. Sequence information provides complementary information that is not fully covered by three-dimensional structure data. This can improve the accuracy of predicting protein–small molecule interactions and protein functional sites [125,128].

## 4. Conclusions and Outlook

At present, intelligent protein design is in a boom period, and several intelligent protein design models were developed, including SCUBA, ABACUS, ProteinMPNN, and RFdiffusion. This significantly improved the success rate and computational efficiency of protein design. However, the accurate and rapid protein design concept of ‘You get what you designed’ is yet to be realized in practice.

Effective protein characterization is essential for intelligent protein design. Four protein characterization methods (namely, traditional descriptor-based, sequence-based, structure-based, and hybrid sequence-structure-based methods), were introduced. Traditional protein representation methods were applied in the early days due to their simplicity and ease of understanding. However, they could not comprehensively represent proteins. The similarity between natural language and protein sequences resulted in sequence-based protein characterization methods based on NLP becoming the main method for protein sequence characterization. Structure-based protein characterization methods, such as point clouds based on spatial coordinates and GVP-GNNs based on geometric vectors, have also received widespread attention with the rapid development of protein structure prediction methods and artificial intelligence algorithms. However, their applications are limited because of their high computational requirements. Researchers have attempted to integrate sequence and structural information to represent proteins to comprehensively consider computational power and protein characterization; however, determining the best combination of multiple features remains still an open question.

Although the representation of proteins for intelligent model construction is largely resolved, there is no consensus on which representation is most appropriate for characterizing proteins. We believe that a large amount of protein structure resolution and the development of intelligent algorithms will inspire new efforts to improve protein characterization. This would promise to accurately extract useful information from the vast amount of data, and associate sequence structure information with functional phenotypes to enable efficient and accurate protein design with new functions.

## Figures and Tables

**Table 1:** Several tools for protein structure prediction derived from AlphaFold2 and RoseTTAFold.

Methods	Models	Inputs	Multimeric Structure	Advantages	URLs	References
ColabFold	JAX	MSA-based	Yes	40–60 × faster prediction than AlphaFold2, and user friendly	https://github.com/sokrypton/ColabFold, accessed on 24 November 2023	[65]
OpenFold	PyTorch	MSA-based	Yes	PyTorch replication of AlphaFold, high flexibility	https://github.com/aqlaboratory/openfold, accessed on 24 November 2023	N/A
Uni-Fold	PyTorch	MSA-based	Yes	Friendly operating environment, and wide hardware adaptation	https://github.com/dptech-corp/Uni-Fold, accessed on 24 November 2023	[66]
FastFold	PyTorch	MSA-based	No	Reduced training time from 11 days to 67 h	https://github.com/hpcaitech/FastFold, accessed on 24 November 2023	[67]
HelixFold	PaddleHelix	MSA-based	No	Improved training and prediction speed, and reduced memory consumption	https://github.com/PaddlePaddle/PaddleHelix/tree/dev/apps/protein_folding/helixfold, accessed on 24 November 2023	[68]
MindSpore-Fold	MindSpore	MSA-based	Yes	Based on MindSpore framework, high performance, and fast prediction speed	https://github.com/mindspore-ai/mindspore, accessed on 24 November 2023	N/A
MEGA-Fold	MindSpore	MSA-based	No	More accurate and efficient protein structure prediction than AlphaFold2	https://gitee.com/mindspore/mindscience/tree/master/MindSPONGE/applications/MEGAProtein, accessed on 24 November 2023	[69]
EMBER3D	PyTorch	pLM-based	No	Ability to visualize the effect of mutations on predicted structures and high predictive efficiency	https://github.com/kWeissenow/EMBER3D, accessed on 24 November 2023	N/A
ESM-Fold	PyTorch	pLM-based	No	Reduced dependence on MSA input, inference speed is an order of magnitude faster than AlphaFold2	N/A	[51]
HelixFold-Single	PaddleHelix	pLM-based	No	Breaking the speed bottleneck of relying on MSA retrieval models, and prediction accuracy is comparable to AlphaFold2 and nearly a thousand times faster	https://github.com/PaddlePaddle/PaddleHelix/tree/dev/apps/protein_folding/helixfold-single, accessed on 24 November 2023	[70]
OmegaFold	PyTorch	pLM-based	No	Protein homology-independent, easy to install, and overall predictive power comparable to AlphaFold2 and RoseTTAFold	https://github.com/HeliXonProtein/OmegaFold, accessed on 24 November 2023	[71]
IgFold	PyTorch	pLM-based	No	Focus on antibody structure prediction, high prediction accuracy, and prediction time less than 1 min	https://github.com/Graylab/IgFold, accessed on 24 November 2023	[72]
D-I-TASSER	PyTorch	MSA-based		Higher prediction accuracy with online server	https://zhanggroup.org/D-I-TASSER/, accessed on 24 November 2023	[73]

**Table 2:** Summary of characteristics, properties, and applications of traditional descriptor characterization methods.

	Encoding	Description	Characteristic	Main Category	Application
Based on the sequence	k-mer	K-mer is a subsequence of length k that is used to minimize the effects of arbitrary starting points, where k is an integer, ranging from 1 to hundreds.	Reflects the frequency of k-conjoined AAs in the protein sequence.	Based on AA information	[125,126,127]
	PSSM	Logarithm of the probability of all possible molecular types occurring at each position in a given biological sequence.	Powerful, but neglects the interactions between different residues.	Based on evolutionary information	[128,129]
	BLOSUM	Reflects the exchange probability of AA pairs.	Research results vary with the type of matrix.	Based on evolutionary information	[130]
	Autocorrelation	The interdependence of AAs in a given sequence.	Reduces the feature space and standardize the sequence length.	Based on physicochemical properties	[131]
	CTD	The composition, transition, and distribution (CTD) of AAs in a given sequence.	Reflects the distribution of AAs with diverse structures and physicochemical characters in a given sequence.	Based on physicochemical properties	[132,133,134]
	CTriad	The conjoint triad (CTriad) is generally regarded to consist of a combination of three adjacent AAs.	AAs were divided into 7 groups based on the side chain volume and dipole.	Based on physicochemical properties	[135,136]
	Z-scales	The Z-scales obtained from the field of quantitative sequence- activity modeling (QSAM).	The most widely used descriptor set in proteochemometric modeling,	Based on physicochemical properties	[137]
	VHSE	Vectors of hydrophobic, steric, and electronic properties (VHSE) are derived from principal components analysis (PCA) of independent families of 18 hydrophobic properties, 17 steric properties, and 15 electronic properties, respectively.	VHSE is of relatively definite physicochemical meaning, easy interpretation, and contains more information compared with z scales.	Based on physicochemical properties	[138,139]
	ProtFP	Protein Fingerprint (ProtFP) is based on a selection of different AA properties obtained from the AAindex database.	The descriptor was obtained using recursive elimination of the most co-varying properties after starting with the full set of indices.	Based on physicochemical properties	[139,140]
	FASGAI	The factor analysis scales of generalized AA information (FASGAI) are derived from 335 physicochemical properties of the 20 natural AAs.	Applying a factor analysis rather than a PCA.	Based on physicochemical properties	[139,141]
Based on the structure	T-scale	Derived from PCA on the 67 kinds of structural and topological variables of 135 AAs.	The 3D properties of each structure are not explicitly considered.	Topology-based representation method	[142]
	ST-scale	Structural topology scale (ST-scale) was recruited as a novel structural topological descriptor derived from PCA on 827 structural variables of 167 AAs.	The molecular structure was optimized, and 3D information of AAs was used.	Topology-based representation method	[143,144]
	MSWHIM	The MSWHIM descriptor set is derived from 36 electrostatic potential properties obtained from the 3D molecule structure.	The number of indicators is simple, easy to calculate, and invariant to the coordinate system.	Geometric-based representation method	[145]

## Data Availability

No new data were created or analyzed in this study.
